# Bilateral Cerebellopontine Angle Masses in a 72-Year-Old Woman With Prior Malignant Melanoma and a Cochlear Implant: A Diagnostic and Management Challenge

**DOI:** 10.7759/cureus.99471

**Published:** 2025-12-17

**Authors:** Syeda Faiza Fatima, Ibidapo Yusuf

**Affiliations:** 1 Department of Emergency Medicine, North West Anglia NHS Foundation Trust, Peterborough, GBR

**Keywords:** brain metastases, cerebellopontine angle, cochlear implant, diagnostic challenge, malignant melanoma, metastatic melanoma, neurological deterioration, palliative care, vestibular schwannoma, visual loss

## Abstract

Cerebellopontine angle masses are most frequently vestibular schwannomas, but may also represent metastatic disease, particularly in patients with a history of malignant melanoma. We describe the case of a 72-year-old woman with a prior history of melanoma and a right cochlear implant who presented with rapidly progressive bilateral hearing loss, visual impairment, imbalance, and right-sided facial weakness. An initial non-contrast CT scan was reported as normal, although a later multidisciplinary review identified bilateral cerebellopontine angle lesions. MRI, the gold standard for characterising lesions of this region, could not be performed because of the cochlear implant. The differential diagnosis included bilateral vestibular schwannomas and metastatic melanoma.

During hospitalisation, the patient developed an infection that resolved with antibiotic therapy but continued to deteriorate neurologically, with worsening visual loss and recurrent falls. A repeat contrast-enhanced CT scan demonstrated enlargement of the cerebellopontine angle lesions with associated cerebral oedema and ventricular dilatation. Her past genetic testing had confirmed BRAF positivity, though her frailty and poor functional status excluded neurosurgical or targeted systemic treatment. Following multidisciplinary and family discussions, palliative care was initiated. The patient experienced progressive decline and died 45 days after admission. This case illustrates the diagnostic and therapeutic challenges associated with bilateral cerebellopontine angle lesions in patients with a history of melanoma when MRI is contraindicated. It highlights the aggressive behaviour of melanoma metastases, the limitations in imaging and treatment options in frail patients with implants, and the importance of involving palliative care early to optimise symptom management and family support.

## Introduction

Cerebellopontine angle lesions are most commonly vestibular schwannomas, followed by meningiomas and epidermoid cysts [1]. Although metastatic disease is less common, melanoma is a recognised but aggressive cause of secondary cerebellopontine angle involvement [2]. MRI remains the preferred imaging modality because it provides superior soft tissue contrast and allows detailed assessment of cranial nerves and adjacent structures [3]. Advances in systemic therapies, including radiotherapy, immunotherapy, and newer targeted approaches, have broadened management options for melanoma brain metastases [4]. However, MRI cannot be performed in certain patients due to implanted devices, which may delay diagnosis and compromise early clinical decision-making.

In individuals with a history of melanoma, cerebellopontine angle masses require careful evaluation of both benign and malignant causes. The radiological overlap between vestibular schwannomas and metastatic melanoma becomes particularly challenging when MRI is contraindicated. This report describes an elderly woman with a history of malignant melanoma and a right cochlear implant who developed bilateral cerebellopontine angle masses with rapid neurological deterioration.

## Case presentation

A 72-year-old woman presented with three to four weeks of progressive bilateral hearing loss, decreasing vision, gait imbalance and right-sided facial weakness. She had been seen twice previously in the emergency department for similar symptoms and was discharged with outpatient otolaryngology and ophthalmology follow-up. She returned because her symptoms continued to worsen.

Her medical history included melanoma of the skin treated by wide local excision, malignant melanoma of the breast and axillary lymph nodes treated with surgical resection followed by nivolumab immunotherapy, a positive BRAF mutation and a right cochlear implant. On examination, she had profound bilateral hearing impairment that required communication through writing. Her right eye visual acuity was limited to finger counting; pupils were equally sized and bilaterally reactive. Gait assessment showed significant unsteadiness and a positive Romberg test. She had a right lower motor neuron facial palsy with exposure keratopathy. No additional focal neurological deficits were identified.

**Figure 1:**
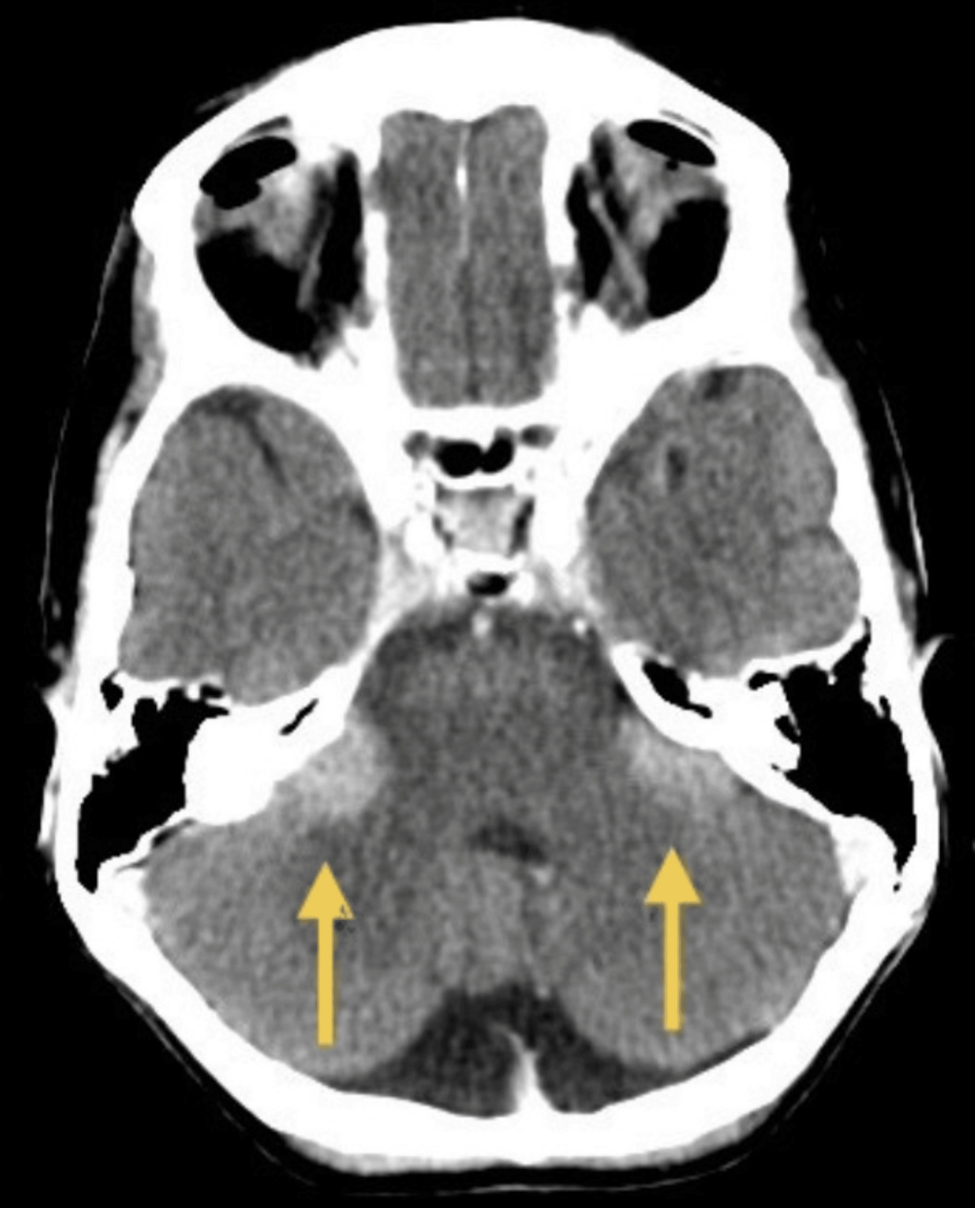
Non-contrast axial CT scan showing a cerebellopontine angle mass on the both sides. This non-contrast CT scan demonstrates a soft tissue mass involving both cerebellopontine angles. The lesion was initially overlooked on the first radiology report and was later identified following multidisciplinary review.

The initial non-contrast CT of the brain was reported as normal. On subsequent multidisciplinary review, a soft tissue abnormality in both cerebellopontine angles was identified (Figure 1). The differential diagnosis included bilateral vestibular schwannomas and metastatic melanoma. MRI could not be performed due to the cochlear implant.

**Figure 2:**
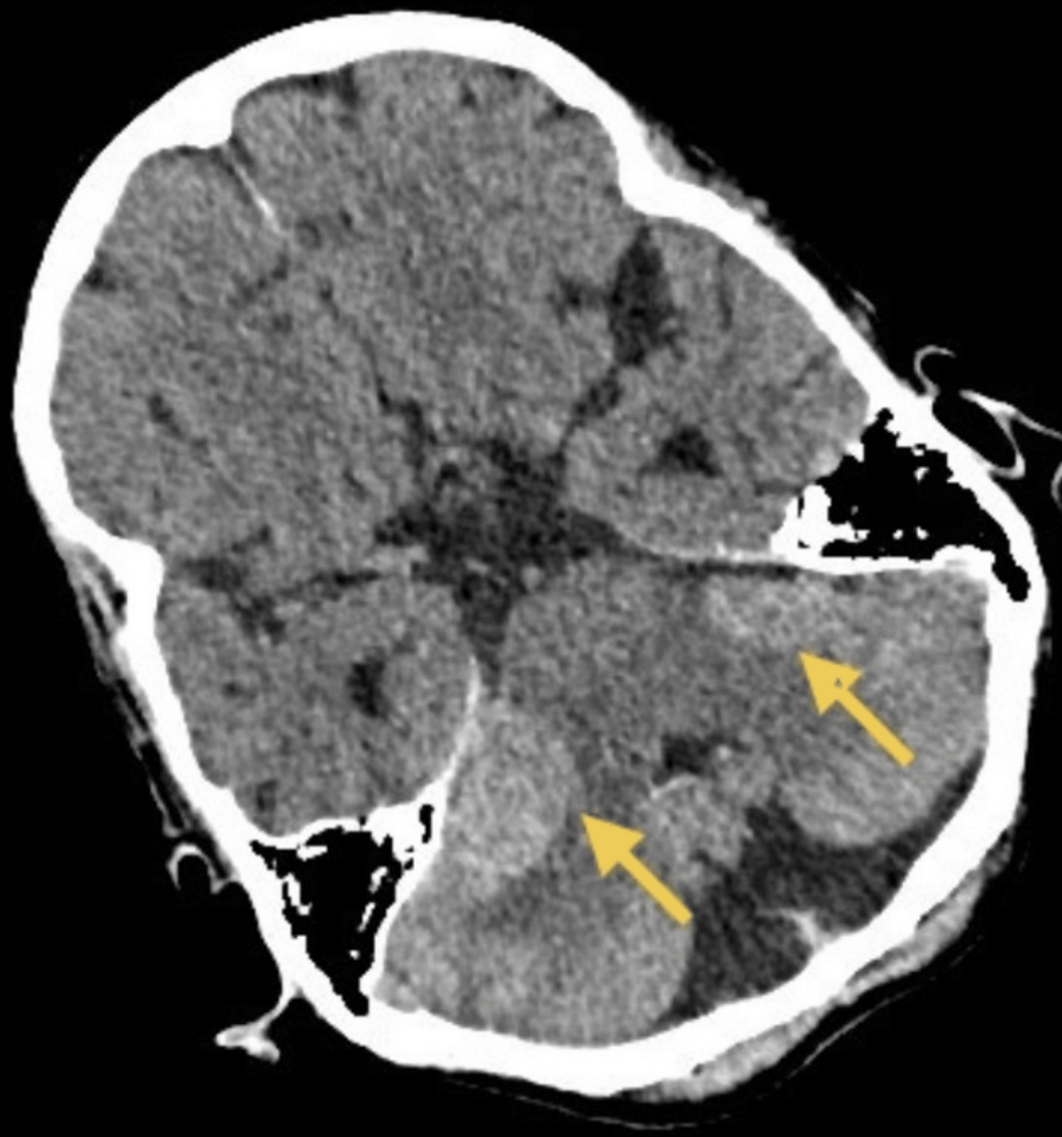
Contrast axial CT scan showing drastic increase in the size of bilateral cerebellopontine angle abnormalities. This contrast-enhanced CT image shows bilateral cerebellopontine angle lesion enlargement with cerebral oedema and ventricular dilatation. These findings contributed to the diagnostic uncertainty in the absence of an MRI.

During admission, the patient developed an infection of unknown origin, demonstrated by raised CRP and WBC without any systemic symptoms, which resolved with broad-spectrum intravenous antibiotics. Despite this, she experienced progressive visual decline and several inpatient falls. After a second fall, a contrast-enhanced CT of the brain demonstrated interval enlargement of the cerebellopontine angle lesions, associated cerebral oedema and new ventricular dilatation (Figure 2). Neurosurgical assessment concluded that she was not suitable for surgical resection or stereotactic radiotherapy. Although BRAF mutation positivity provided a potential therapeutic pathway with BRAF inhibitors (like dabrafenib and vemurafenib) and MEK inhibitors (like trametinib) that block this specific pathway, slowing tumour growth, her frailty and rapid deterioration excluded systemic treatment. Following family discussions and multidisciplinary agreement, an AMBER (Assessment, Management, Best practice, Engagement, Recovery uncertain) care bundle was initiated [5] that involves a systematic approach for hospital inpatients facing unclear recovery from acute illness. Her condition continued to worsen, and she died 45 days after admission.

## Discussion

The diagnostic complexity in this case highlights the challenges that arise when standard neuroimaging pathways cannot be followed. MRI is central to characterising cerebellopontine angle pathology because of its superior soft-tissue resolution and its ability to visualise the cranial nerves in detail [1,3,6]. When MRI is contraindicated, such as in patients with older cochlear implants, CT becomes the primary modality. CT lacks the sensitivity required for posterior fossa assessment [1,3], and early posterior fossa lesions may be missed, as reflected in the initially normal CT scan [3]. With symptom progression, multidisciplinary reassessment enabled the eventual identification of bilateral lesions. This emphasises the importance of correlating radiological findings with evolving clinical features rather than relying solely on initial imaging impressions [7].

The bilateral cerebellopontine angle involvement further complicated the diagnosis. Bilateral vestibular schwannomas are characteristic of neurofibromatosis type 2, although this diagnosis was unlikely in this patient because of the absence of typical clinical features and the rapid progression of neurological decline [1]. The clinical presentation, which included hearing loss, facial nerve palsy, visual impairment, and truncal instability, was more consistent with an aggressive and infiltrative process. Published cases have described similar diagnostic difficulties in malignant cerebellopontine lesions presenting with progressive unilateral or bilateral hearing loss. These reports highlight how difficult it can be to distinguish malignant from benign pathology in this region [8]. Metastatic melanoma can mimic benign cerebellopontine angle tumours, particularly when imaging is limited to CT [2,7]. The interval enlargement and oedema seen on follow-up CT strengthened the suspicion of malignant involvement [2,3], which reflects broader analyses describing the radiological heterogeneity of malignant cerebellopontine angle tumours [9]. This case illustrates how diagnostic uncertainty is amplified in patients with an oncological history and restricted imaging options [7].

Management decisions were shaped by the patient’s frailty and rapid neurological deterioration. Systemic treatments, including immunotherapy and BRAF-targeted therapy, have shown improved outcomes in selected patients with melanoma brain metastases [4,6]. These therapies require sufficient physiological reserve to tolerate potential adverse effects. In this case, the pace of clinical decline precluded their use [4]. This highlights the principle that management strategies must reflect real-time clinical status rather than theoretical eligibility for treatment [4,6].

Overall, this case highlights several broader implications for clinical practice. Clinicians should maintain a high index of suspicion for malignant pathology when patients with a history of melanoma present with atypical or rapidly evolving cerebellopontine angle symptoms, even when initial imaging appears unremarkable [2,7]. When MRI is unavailable, close clinical monitoring, repeated imaging, and multidisciplinary review are essential to limit diagnostic delays [1,3]. Early discussions regarding goals of care [5] are also important when diagnostic uncertainty coexists with clinical decline, ensuring that management aligns with prognosis and patient priorities [8].

## Conclusions

This case highlights the significant diagnostic and management challenges associated with cerebellopontine angle masses in patients with a history of malignant melanoma, particularly when MRI cannot be performed due to implanted devices. The rapid clinical deterioration and limitations in diagnostic clarity show how melanoma metastases can present atypically and progress aggressively. The inability to offer surgical or targeted therapeutic interventions due to the patient’s frailty further underscores the importance of aligning treatment decisions with functional status rather than theoretical eligibility. This case also emphasises the need for multidisciplinary collaboration, timely clinical reassessment, and realistic expectations regarding outcomes in similar presentations. Importantly, it demonstrates the value of early integration of palliative care when curative or disease-modifying options are no longer appropriate.

## References

[1] Samii M, Matthies C (1997). Management of 1000 vestibular schwannomas (acoustic neuromas): surgical management and results with an emphasis on complications and how to avoid them. Neurosurgery.

[2] Xie HM, Li L, Richard SA, Lan Z, Zhang Y (2022). Cerebellopontine angle metastatic melanoma mimicking schwannoma in a sexagenarian: case report. Adv Biosci Clin Med.

[3] Radder N, Radder SB (2025). Imaging in cerebellopontine angle masses. Int J All Res Educ Sci Methods.

[4] Long GV, Trefzer U, Davies MA (2012). Dabrafenib in patients with Val600Glu or Val600Lys BRAF-mutant melanoma metastatic to the brain (BREAK-MB): a multicentre, open-label, phase 2 trial. Lancet Oncol.

[5] (2025). Transforming end of life care in acute hospitals. https://www.england.nhs.uk/wp-content/uploads/2016/01/transforming-end-of-life-care-acute-hospitals.pdf.

[6] Tawbi HA, Forsyth PA, Algazi A (2018). Combined nivolumab and ipilimumab in melanoma metastatic to the brain. N Engl J Med.

[7] Marzolino R, Castro V, Gambacorta V, Tonon E, Cattaruzzi E, Orzan E (2024). A case report of malignant cerebellopontine angle lesion highlighting the interdisciplinary diagnostic challenge in the case of unilateral progressive hearing loss. J Clin Med.

[8] Shinogami M, Yamasoba T, Sasaki T (1998). Bilateral isolated metastases of malignant melanoma to the cerebellopontine angle. Otolaryngol Head Neck Surg.

[9] Eliezer M, Tran H, Inagaki A (2019). Clinical and radiological characteristics of malignant tumors located to the cerebellopontine angle and/or internal acoustic meatus. Otol Neurotol.

